# Use of Free, Open Access Medical Education and Perceived Emergency Medicine Educational Needs Among Rural Physicians in Southwestern Ontario

**DOI:** 10.7759/cureus.796

**Published:** 2016-09-21

**Authors:** Alex Folkl, Teresa Chan, Elaine Blau

**Affiliations:** 1 Emergency Medicine, Guelph General Hospital; 2 Faculty of Health Sciences, Division of Emergency Medicine, McMaster University; 3 Department of Family Medicine - Rural Residency Site Director, McMaster University

**Keywords:** emergency medicine, medical education, rural medicine, family medicine

## Abstract

Free, open access medical education (FOAM) has the potential to revolutionize continuing medical education, particularly for rural physicians who practice emergency medicine (EM) as part of a generalist practice. However, there has been little study of rural physicians’ educational needs since the advent of FOAM. We asked how rural physicians in Southwestern Ontario obtained their continuing EM education. We asked them to assess their perceived level of comfort in different areas of EM. To understand how FOAM resources might serve the rural EM community, we compared their responses with urban emergency physicians. Responses were collected via survey and interview. There was no significant difference between groups in reported use of FOAM resources. However, there was a significant difference between rural and urban physicians’ perceived level of EM knowledge, with urban physicians reporting a higher degree of confidence for most knowledge categories, particularly those related to critical care and rare procedures. This study provides the first description of EM knowledge and FOAM resource utilization among rural physicians in Southwestern Ontario. It also highlights an area of educational need -- that is, critical care and rare procedures. Future work should address whether rural physicians are using FOAM specifically to improve their critical care and procedural knowledge. As well, because of the generalist nature of rural practice, future work should clarify whether there is an opportunity cost to rural physicians’ knowledge of other clinical domains if they chose to focus more time on continuing education in critical care EM.

## Introduction

Free, open access medical education (FOAM) consists of medical curriculum delivered over the internet, for free, and is accessible anytime. It has the potential to revolutionize continuing education in emergency medicine (EM) [1].

One group that may benefit significantly from FOAM is rural physicians who practice emergency medicine as part of a generalist practice (referred to as rural emergency physicians, or rural EPs, in this report, in contrast with their urban counterparts). Many of these physicians practice a broad scope of medicine, which often includes EM alongside outpatient and inpatient medicine. Time constraints associated with these other clinical duties may make it difficult for these physicians to attend EM conferences for skills updates and to sift through the extensive EM literature to identify new relevant research. These factors combined may make rural EPs less comfortable than their urban colleagues in caring for critically ill patients and in search of ongoing EM education [2-3]. However, critically ill patients do present to rural emergency departments (EDs). For example, in 2009, 6.9% of patients presenting to rural Ontario EDs required immediate resuscitation or evaluation within 15 minutes [4]. It is, therefore, necessary that rural EPs be knowledgeable in caring for critically-ill patients.

FOAM may offer an opportunity to improve rural EPs’ comfort in caring for critically-ill patients because it is accessible from the rural environment and a significant proportion of its content is devoted to the resuscitation of critically ill patients. However, there has been very little study of rural EP’s educational needs since the advent of FOAM.

We asked how rural EPs in Southwestern Ontario obtained their continuing EM education. We asked them to assess their perceived level of comfort in areas of EM, with a focus on critical care. To better understand how FOAM resources might serve the rural EM community, we compared their responses with urban EPs. Responses were collected via survey and interview.

## Materials and methods

### Survey design and distribution

One study co-author (AF) developed an EM Knowledge and Educational Needs Assessment Survey, which can be seen in the Appendix. This survey was piloted upon and refined with feedback from the other co-authors (TC and EB). The survey was emailed to rural and urban EPs affiliated with McMaster University in Southwestern Ontario. A convenience sample of survey responses was collected from January 28th to February 23rd, 2015. Informed consent was obtained from participants prior to participation.

### Statistics

Survey statistics were computed with Statistical Package for Social Sciences (SPSS) (IBM SPSS Statistics, Armonk, NY). Chi-squared analysis was used to compare the EPs’ use of online EM resources, and the Kruskal-Wallis test was used to compare the two populations of EPs’ relative comfort in different domains of EM knowledge.

### Interview design and analysis

Survey respondents were recruited to participate in a focus group to explore issues raised in the initial survey. Informed consent was obtained from participants prior to participation. Only three survey respondents agreed to participate; therefore, our protocol was changed slightly at this stage, and interviews, rather than a focus group, were conducted with respondents. Interviews were held over Google Hangouts. They consisted of scripted and unscripted questions. They were recorded, transcribed, and analyzed to identify themes in the interviewees’ responses by one study co-author (AF). Themes were used to triangulate survey data.

This study received ethics approval from the Hamilton Integrated Research Ethics Board, approval #15-013.

## Results

### Demographics and practice patterns

Thirty-eight physicians completed the survey. Rural EPs were slightly older than urban EPs and had been in practice longer, worked fewer hours per week in the ED, and EM comprised a smaller percentage of their clinical work week. All urban EPs and 16% of rural EPs had completed residency-based training in EM. Twenty-six percent of rural EPs and 63% of urban EPs reported completing additional, formalized post-residency EM courses, such as Advanced Trauma Life Support (ATLS). However, it is likely this reflects underreporting as at least one advanced life support course, Advanced Cardiovascular Life Support (ACLS), is mandatory for hospital privileges across the province, and in at least two locations included in our survey, ATLS is required as well (Table 1).

**Table 1:** Survey Respondent Demographics IQR = interquartile range; EM = emergency medicine

	Age (Median, 25%-75% IQR)	Years in Practice (Median, 25%-75% IQR)	Weekly EM Hours (Median, 25%-75% IQR)	EM Hours as Percent of Clinical Work Week (Median, 25%-75% IQR)	Completion of Formal EM Residency Training Program (%)	Completion of EM Continuing Education Courses (%)
Rural	45, 34-37	17, 8.25-20	12, 4.5-20	20%, 5-25	16	26
Urban	41, 35.5-46.5	11, 7.5-18	32, 24.5-39	90%, 65-97.5	100	63

Rural respondents practiced in six communities across southwestern Ontario, ranging in population from approximately 1,000 to approximately 22,000. Only three rural respondents practiced in locations identified as “rural” by either the Ontario Ministry of Health’s Rurality Index or Statistics Canada, but all practiced in sites affiliated with McMaster University’s Rural Stream Family Medicine residency program. For purposes of data analysis, we used McMaster’s definition of “rural.” Urban respondents practiced in three communities in Southwestern Ontario, ranging in population from approximately 366,000 to approximately 713,000.

The division of extra-EM duties was also different between rural and urban EPs. Rural EPs also worked in family practice clinics, provided in-patient care, obstetrical care, or anesthesia while urban EPs also worked as researchers, trauma team leaders, or intensivists. Many rural and urban EPs contributed to undergraduate and postgraduate medical education.

### Emergency medicine education

Rural and urban EPs reported a similar use of EM resources, with the exception of Twitter and Facebook, which urban physicians used more frequently. Urban EPs were also more likely to use EM resources to connect with other EPs. There was no significant difference between groups in reported use of FOAM resources (Table 2).

**Table 2:** Percentage of Rural and Urban EPs Using Selected Online EM Resources

Resource	Rural (%)	Urban (%)	P value (Chi-square)
Twitter	5	36	0.02
Facebook	13	50	0.02
Google Plus	39	29	0.72
eTexts	70	71	1
Podcasts	61	71	0.72
Vodcasts	39	50	0.73
Blogs	22	43	0.27
Wikis	17	43	0.13

### Emergency medicine knowledge deficits

There was a consistent, significant difference between how rural and urban EPs perceived the level of EM knowledge, with urban EPs reporting a higher degree of confidence for most knowledge categories (Table 3). Many of these categories were in areas of critical care.

**Table 3:** Self-reported Confidence of Rural and Urban EPs in Selected EM Domains on a Five-point Likert Scale 5= most confident; 1 = least confident EP = emergency physicians; EM = emergency medicine; IQR = interquartile range

EM Domain	Rural (Median, 25%-75% IQR)	Urban (Median, 25%-75% IQR)	P value
EM Knowledge	3, 3-4	5, 4-5	< 0.01
EM Procedures	3, 3-4	4, 4-5	< 0.01
Adult resuscitation	4, 4-4	5, 5-5	< 0.01
Pediatric resuscitation	3, 3-3	4, 3.5-4.5	0.02
Cardiac emergencies	4, 4-4	5, 5-5	< 0.01
Respiratory emergencies	4, 4-5	5, 5-5	< 0.01
Gastrointestinal emergencies	4, 3-4	5, 4-5	0.02
Trauma	3, 3-4	5, 4-5	0.03
Neurologic emergencies	3, 3-4	5, 5-5	< 0.01
Psychiatric emergencies	4, 4-4	5, 4-5	0.04
Sepsis	4, 4-4	5, 5-5	< 0.01
Toxicology	3, 2-3	4, 4-5	< 0.01
Intraosseous insertion	4, 3-4	5, 4.5-5	0.1
Intubation	3, 3-4.5	5, 5-5	0.01
Casting	4, 4-5	5, 5-5	0.03
Suturing	5, 5-5	5, 5-5	0.03
Procedural sedation	3, 3-4	5, 5-5	< 0.01
Central line placement	2, 1.5-3	5, 4-5	0.01
Joint aspiration	4, 3.5-5	5, 4-5	0.03
Dislocation reduction	4, 3-4.5	5, 5-5	< 0.01
Chest tube placement	3, 2-5	5, 4.5-5	0.01
Cardioversion or defibrillation	4, 3-5	5, 5-5	< 0.01
EKG interpretation	4, 4-5	5, 4-5	0.05
Chest xray interpretation	4, 4-4	5, 4-5	0.02
Minor surgical procedures	4, 4-5	5, 5-5	< 0.01
Team leadership	4, 4-5	5, 4.5-5	< 0.01
Conflict resolution	4, 3.5-5	4, 4-5	0.29
Continuous quality improvement	4, 3-4	4, 4-4.5	0.15

Three rural EPs agreed to participate in interviews (M = 1). All practiced a broad spectrum of rural medicine, with a mix of family practice, hospitalist, obstetrics, and EM. Two had been in practice for less than two years; one had been practicing for 25 years.

### Emergency medicine education

Interviewees used a wide variety of resources for EM education, including FOAM (Academic Life in Emergency Medicine, Life in the Fast Lane) and non-FOAM (Up To Date, Google). All found that the resources they preferred were succinct and easily searchable. One interviewee suggested there was not enough rurally-focused FOAM. From the survey data, we found that some participants were overwhelmed by the volume of online education but also suggested that perhaps not all the online education was of the same quality and not as relevant to their practice:

"Too many sites...Often opinion lead. Frequently produced in [anotther] country where practice and drugs differ. I often get the feeling that the producers are staging the work of the 'friends' rather than giving an unbiased view of treatment / management options." (Survey respondent)

### Emergency medicine knowledge deficits

All interviewees found that rare procedures, particularly those that had come up very seldom in residency or post-residency training courses, comprised the majority of their EM knowledge deficit. Bedside ultrasound, shoulder reductions, and chest tubes were specifically mentioned. Interviewees had attempted to gain competency in these procedures in a variety of ways, including watching YouTube videos, advocating for enhanced residency training, and looking for official courses, without success. One respondent noted:

“Simulation would have been real helpful. That was something kept trying to get going, but nobody else was in on it.”

Another physician had looked for a course in Emergency Department Echo but stated: “I only know one physician in the area who's not even in the Emergency Department all the time who can sign… off” independent scans, making obtaining independent practitioner status quite difficult.

## Discussion

Rural EPs in Southwestern Ontario are using FOAM resources alongside traditional EM resources for their EM education. They identify a significantly lower level of comfort with critical care knowledge and procedures compared with urban EPs. It is possible that this reduced level of comfort comes from a decreased frequency of treating critically ill patients.  

It is also possible that maintaining a broad set of skills required to practice in a rural environment means that rural EPs, as rural generalists, are by necessity balancing their continuing education time in critical care emergency medicine with continuing education in areas of obstetrics, inpatient medicine, outpatient medicine, palliative care, FP-anesthesia, and long-term care.

Considering our findings, however, it is worthwhile to note that recent literature around the nature of the coverage of topics by FOAM outlets has suggested that topics including airway techniques, ECG interpretation, interpretation of recent literature, resuscitation, ultrasonography were deemed over-represented [6]. Curiously, these topics are the topics which we identified as the most important needs for rural EPs compared to their urban peers. As such, it may be useful to develop guides for rural EPs to introduce them to FOAM sites that may be of interest to them. A useful starting point may be the list of top sites as ranked by the* Social Media Index *(https://www.aliem.com/social-media-index/), hosted at the *Academic Life in Emergency Medicine* site. This ranking site has been described in the literature by Thoma, et al. as a potential way of ranking the most followed sites [7]. This may serve as a good starting point for rural EPs looking for further education on key procedural and resuscitation topics. There are also other introductory primers within the literature that may be of use to physicians new to online education [8].  

Our results suggested that rural EPs are keenly aware that online education products may be of variable quality. Recent innovations, such as blog-based peer review procedures [9-10] and quality appraisal tools designed specifically for evaluating medical education blogs (e.g., the ALiEM AIR score and the METRIQ-5/METRIQ-8 scores) [11-12], will be of interest to this group as well as their urban/academic colleagues. Using these quality appraisal tools, some educators are creating guides to FOAM posts [13], which are being published in traditional literature to bridge the divide between traditional and online resources.

### Future directions

As identified by some of our participants, judging quality in the world of FOAM can be difficult. Although some quality appraisal tools have been created [11-12], it is unclear whether our study's target population will find these scoring systems acceptable or useful. Rural EPs may be, in fact, a distinct population worth engaging when developing or validating these appraisal tools.

It is important to note, however, that online education (and FOAM, specifically) may not be the best way to learn all skill gaps identified in our present needs assessment. There is no clear evidence that online videos can be used as a pure substitution for other more traditional methods of learning (e.g., simulation settings or workshops). Future studies may be worth conducting to see if online preparation with practicing rural EPs might enhance or help to maintain procedural skills.

Future work might also clarify whether there is an opportunity cost to rural EPs’ knowledge of other clinical domains if they chose to focus more time on continuing education in critical care emergency medicine.

### Limitations

Generalizability of these results is limited by several factors. First, our sample size was small, and our survey had an unknown response rate since we used a snowball sampling technique and ask participants to email to their local groups that comprised of mixed practices (rural EPs, family physicians, rural anesthetists). It may also have suffered from selection bias: We recruited participants via email to complete a survey about online resource utilization, which was administered via the internet. EPs less inclined to use online or FOAM resources may have chosen not to participate. Second, a definition of “formal training” in EM was not provided to survey respondents, which, as noted above, may have led to underreporting of standard resuscitation courses required by all hospitals for EM privileges. Third, it is unknown whether rural EPs' lower confidence in critical care has any effect on patient outcomes. Prior studies have demonstrated a poor correlation between self-assessment and objective measures of competence [5]. Finally, as noted above, only three survey respondents were “rural,” when classified by Rurality Index or Statistics Canada’s definition of the term.

## Conclusions

This study provides the first description of EM knowledge and FOAM resource utilization among rural EPs in Southwestern Ontario. It also highlights an area of educational need -- that is, critical care and rare procedures. Future work should address whether rural EPs are using FOAM specifically to improve their critical care and procedural knowledge, determine how existing FOAM might be tailored to better reflect the realities of rural EM practice, and should assess FOAM more directly, alongside traditional methods of continuing medical education, such as textbooks and conferences. Future work on FOAM should include practitioners of rural EM since they have needs that are disparate from urban physicians.

## Appendices

Appendix:  QA EM Survey

1. What is your age?

2. What is your gender?

3. How many years have you been practicing medicine?

4. What city or town do you practice medicine in the most?

5. In an average week, how many hours do you work in an emergency department?

6. What percentage of your total clinical time per week is spent working in an emergency department?

7. Have you completed formal training in emergency medicine outside of required residency rotations?

8. If yes, what formal training did you complete?

9. Have you obtained a formal emergency medicine designation? (e.g. CCFP-EM or Royal College)

10. In general, how confident are you in your level of emergency medicine-specific clinical knowledge? (**Likert scale, 1-5**)

11. In general, how confident are you in your level of emergency medicine-specific procedural competency? (**Likert scale, 1-5)**

12. Please rate your knowledge of the following areas of emergency medicine (**Likert Scale, 1-5)**

12.1. Adult resuscitation

12.2. Pediatric resuscitation

12.3. Cardiac emergencies (ACS, arrythmias, etc)

12.4. Pulmonary emergencies (asthma, COPD, etc)

12.5. Gastrointestinal emergencies (bleeding, liver failure, etc.)

12.6. Trauma resuscitation

12.7. Neurologic emergencies (head trauma, stroke)

12.8. Sepsis

12.9. Toxicology

13. Please rate your comfort level in the following emergency medicine procedures (**Likert Scale 1-5)**

13.1. Intubation

13.2. Procedural sedation

13.3. Central line placement

13.4. Chest tube placement

13.5. Cardioversion/defibrillation

13.6. EKG interpretation

13.7. Team leadership

13.8. Conflict resolution

13.9. Continuous quality improvement

14. I use the following for LEARNING/ PROFESSIONAL/ ACADEMIC/ MEDICAL/ PATIENT CARE purposes (Never, Monthly, Weekly or Daily)

- Twitter

- Facebook

- Google+

- eTextbooks

- Podcasts (eg. EMCrit)

- Video Podcasts

- Medical Blogs

- Wikis

- Online File Sharing

- Online Question and Answer Sites

- Other

15. Please rate your relative level of comfort utilizing online resources for CME.

I use free online resources (**Likert 1-5)**

- For general EM education

- To learn about procedures or variations in technique

- To learn interpretive skills

- To be directed to primary literature

- To investigate controversial areas of EM unresolved in core texts

- To answer a question while providing care for an ED patient

- To connect with other EM trainees

- To connect with other EM educators

- To connect with peers in EM and related fields

16. Please rank factors that influence your choice of free online resources

(Not important, Minimal importance, Neutral, Important, Very important)

Clearly identifiable author and credentials

References are provided

Resource that undergoes formal pre-publication peer-review

Authors provide unbiased content, citing opinions other than their own when appropriate

Content is “cutting edge”/innovative

Authors use principles of evidence-based medicine

Entertainment or “Edu-tainment” value of resource

A peer referred you to the resource

A faculty member referred you to the resource

Ease of access

Other

17. Please rank the top three of the following resources in terms of their overall contribution to your EM education (1, 2, 3):

- Textbook

- eTextbook

- Subscription-based resource

- Primary Literature

- Podcast or Video -Podcast

- Medical Blog

- Wiki

- Other

18. Please rate your overall level of confidence in the reliability of online CME resources

19. Please describe barriers to utilization of traditional CME resources, including textbooks, papers, in-person lectures, and in-person courses

20. Please describe barriers to utilization of online CME resources
